# Risk-factor analysis and predictive-model development of acute kidney injury in inpatients administered cefoperazone-sulbactam sodium and mezlocillin-sulbactam sodium: a single-center retrospective study

**DOI:** 10.3389/fphar.2023.1170987

**Published:** 2023-06-08

**Authors:** Ruiqiu Zhang, Liming Gao, Ping Chen, Weiguo Liu, Xin Huang, Xiao Li

**Affiliations:** ^1^ Department of Clinical Pharmacy, The First Affiliated Hospital of Shandong First Medical University and Shandong Provincial Qianfoshan Hospital, Shandong Engineering and Technology Research Center for Pediatric Drug Development, Shandong Medicine and Health Key Laboratory of Clinical Pharmacy, Jinan, China; ^2^ Department of Nephrology, The First Affiliated Hospital of Shandong First Medical University and Shandong Provincial Qianfoshan Hospital, Jinan, China

**Keywords:** cefoperazone-sulbactam sodium, mezlocillin-sulbactam sodium, acute kidney injury, pharmacoepidemiology, risk factors, logic regression model

## Abstract

**Objective:** Acute kidney injury (AKI) is a common adverse reaction observed with the clinical use of cefoperazone-sulbactam sodium and mezlocillin-sulbactam sodium. Based upon real-world data, we will herein determine the risk factors associated with AKI in inpatients after receipt of these antimicrobial drugs, and we will develop predictive models to assess the risk of AKI.

**Methods:** Data from all adult inpatients who used cefoperazone-sulbactam sodium and mezlocillin-sulbactam sodium at the First Affiliated Hospital of Shandong First Medical University between January 2018 and December 2020 were analyzed retrospectively. The data were collected through the inpatient electronic medical record (EMR) system and included general information, clinical diagnosis, and underlying diseases, and logistic regression was exploited to develop predictive models for the risk of AKI. The training of the model strictly adopted 10-fold cross-validation to validate its accuracy, and model performance was evaluated employing receiver operating characteristic (ROC) curves and the areas under the curve (AUCs).

**Results:** This retrospective study comprised a total of 8767 patients using cefoperazone-sulbactam sodium, of whom 1116 developed AKI after using the drug, for an incidence of 12.73%. A total of 2887 individuals used mezlocillin-sulbactam sodium, of whom 265 developed AKI after receiving the drug, for an incidence of 9.18%. In the cohort administered cefoperazone-sulbactam sodium, 20 predictive factors (*p* < 0.05) were applied in constructing our logistic predictive model, and the AUC of the predictive model was 0.83 (95% CI, 0.82–0.84). In the cohort comprising mezlocillin-sulbactam sodium use, nine predictive factors were determined by multivariate analysis (*p* < 0.05), and the AUC of the predictive model was 0.74 (95% CI, 0.71–0.77).

**Conclusion:** The incidence of AKI induced by cefoperazone-sulbactam sodium and mezlocillin-sulbactam sodium in hospitalized patients may be related to the combined treatment of multiple nephrotoxic drugs and a past history of chronic kidney disease. The AKI-predictive model based on logistic regression showed favorable performance in predicting the AKI of adult in patients who received cefoperazone-sulbactam sodium or mezlocillin-sulbactam sodium.

## 1 Introduction

Acute kidney injury (AKI) is a common and complex kidney disease in clinical practice, is associated with poor patient prognosis (Belavgeni et al., 2020), and portends severe consequences (Palevsky, 2006), including increased mortality (Clermont et al., 2002; Thakar et al., 2005). AKI is very common among hospitalized patients, with an incidence in adult patients of 0.7%–77% (James et al., 2010; Han et al., 2013). In addition, it is reported that AKI results in a high mortality of between 14% and 60% in hospitalized adults (Uchino et al., 2005; Bihorac et al., 2013; Chang et al., 2015).

There are over 200 types of clinical medications that induce AKI as observed from investigations of drug-induced AKI (Osman et al., 2017). In addition, among various nephrotoxic drugs that cause AKI, the proportion consisting of antibiotic drugs ranks in the top few percent, of which β-lactam antibiotics accounted for 12.24% of the total. Cephalosporins account for 93.04% of all β-lactam drugs that cause AKI, and their metabolites tend to accumulate in cells as they are excreted by the kidney, resulting in nephrotoxicity. On the other hand, β-Lactam antibiotics or their metabolites can be used as haptens to combine with macromolecular substances *in vivo* to become antigens (Al-Harbi et al., 2003). As a result, the body will produce antibodies, undergo immune reactions, and damage the kidney. Moreover, some authors had confirmed through animal experiments that the nephrotoxicity of cefoperazone-sulbactam sodium was mainly manifested by a large amount of focal necrosis and calcification in the kidney proximal tubule epithelium, as well as the presence of protein and granular tubules in the collecting duct (Erfurth and Hoff, 2000). The second-most prevalent drug used is penicillin, which generally exerts no direct nephrotoxicity but generates acute interstitial nephritis and then acute kidney injury due to allergic reactions (Capelletti et al., 2020). However, there are relatively few studies that focus on risk-factor analysis of AKI in inpatients, particularly in Asian populations. In the present study, we focused on an unbiased estimate of the incidence of AKI in hospitalized patients who received cefoperazone-sulbactam sodium and mezlocillin-sulbactam sodium according to the Kidney Disease: Improving Global Outcomes (KDIGO) definition. In addition, we attempted to analyze the relevant risk factors and develop logistic regression models for AKI vis-à-vis these drugs on the basis of real-world data from our center.

## 2 Materials and methods

### 2.1 Ethical approval

This study was approved by the Ethics Committee of the First Affiliated Hospital of Shandong First Medical University (approval no. YXLL-KY-2022-024), and we did not use personally identifiable information in the present study.

### 2.2 Data sources and searches

The current data were extracted from the electronic medical records of the First Affiliated Hospital of Shandong First Medical University. The collected patient information primarily included the social and demographic characteristics of patients, comorbidities, length of stay, hospitalization expenses, laboratory examination results, medical advice, and diagnosis. We included patients who were discharged from the hospital between 1 January 2018, and 31 December 2020, and who underwent treatment with cefoperazone-sulbactam sodium and mezlocillin-sulbactam sodium (details on the methodology concerning inclusion and exclusion criteria can be found in the Supplementary Material).

### 2.3 Case definition

Our AKI cases were defined in accordance with the 2012 KDIGO Clinical Practice Guidelines for AKI (Kellum et al., 2013), and the diagnostic criteria were shown in Supplementary Table S1. As continuous urine volume monitoring was difficult to perform during the hospitalization of patients in the present study, we based the screening of AKI patients chiefly on the relevant criteria with respect to serum creatinine (sCr) in the AKI diagnostic criteria published by KDIGO. In addition, to reduce missed diagnoses caused by an inability to detect creatinine or a delay in creatinine-detection time and to fully evaluate the correlation between AKI and the use of the target drug and increase the accuracy of the determination of drug-induced AKI for the target drug—we determined the actual AKI diagnosis process in this study based on the AKI clinical practice guidelines published by KDIGO.

According to KDIGO guidelines and combined with the use of target drugs, those patients who met one of the following conditions were determined as having AKI. 1) Patients during hospitalization with β-lactam drugs were used as a starting point, with the most recent sCr value before the first use of the target drug during the patient’s hospitalization and all measured sCr values before discharge used as target data. If the most recent value was determined within the continuous 48-h observation period where the creatinine samples were sorted from front-to-back according to the acceptance date, and the maximal change in the later test value was ≥26.5 compared with any previous test value in μ mol/L (≥0.3 mg/dl), the value was considered to meet the AKI judgment standard. 2) When hospitalization and β-lactam drug administration were used as a starting point, all the most recently measured sCr values before the first use of the target drug during the patient’s hospitalization and before discharge were used as the target data. It is known or assumed that functional kidney damage occurs within 7 days. From the most recent value (if within the continuous observation period of 7 days), the creatinine samples were sorted from front-to-back according to the acceptance date, and if the ratio between the latest detection value and any forward detection value was ≥1.5, it was considered to meet the AKI judgment standard.

We excluded the patients diagnosed with AKI before admission and took into account the AKI caused by other risk factors during the hospitalization of patients before using the study drug. The inpatients with AKI after using β-lactam drugs were then also included.

### 2.4 Outcomes

In this study, we analyzed the incidence of AKI and potential risk factors in hospitalized patients who received cefoperazone-sulbactam sodium and mezlocillin-sulbactam sodium and developed predictive models for AKI risk for inpatients who underwent these drug treatments.

### 2.5 Statistical analyses

We conducted data analysis using R software (version 3.6.3) and implemented the use of big data platforms for healthcare at the First Affiliated Hospital of Shandong First Medical University. Our research variable was designated the independent variable, the AKI group was used as the dependent variable, and the Chi-squared test was applied for univariate analysis. Those variables with *p* < 0.05 in the univariate analysis were then included in multivariate logistic regression analysis. We expressed the analytical results as odds ratios (ORs) and 95% confidence intervals (95% CIs). All *p* values were bilateral, and a *p* value < 0.05 was considered statistically significant. Details regarding our statistical analyses are depicted in Supplementary Material.

### 2.6 Model development

In the present study, the logistic regression models were developed with routinely collected patient information. Logistic regression is the result of linear regression performed by bending the sigmod function. It predicts the probability of a result with only two values. Prediction is based on the use of one or more predictive variables (numerical and categorical). Logistic regression uses maximum likelihood estimation to obtain model coefficients that relate predictive factors to the target. After the initial function estimation, repeat the process until the logarithmic likelihood does not significantly change. Due to the ability to calculate the correlation coefficient between the prediction factor and the target, the model is easy to understand and discover relevant factors.

All predictions with respect to AKI are based on routinely collected patient information, and there were no missing values in the variables required by the AKI predictor. Since no external validation dataset was obtained, we herein adopted a strict 10-fold cross-validation (CV) to test the validity of our model. We employed receiver operating characteristic (ROC) curves and areas under the ROC curves (AUCs) when evaluating the performance of the model to assess its predictive performance. It is conventionally recognized that patients who develop AKI represent only a minority of all hospitalized patients, and the data set used for the present study reflected a great imbalance between positive and negative cases. The presence of a positive/negative case imbalance thus generated the few categories upon which we focused and that were typically ignored by predictive models.

## 3 Results

### 3.1 Demographic characteristics of AKI

We included a total of 8,767 inpatients using cefoperazone-sulbactam sodium. Of these individuals, there were 1,116 cases who met the AKI criteria of the KDIGO 2012 guidelines, suggesting that the incidence of AKI in patients who received cefoperazone-sulbactam sodium was 12.73% (1116/8767). After exclusions, there were 2,887 patients included in the mezlocillin-sulbactam sodium cohort, and of these, 265 patients were diagnosed as AKI cases, with an incidence of 9.18% (265/2887).

The patient characteristics for the cefoperazone sulbactam sodium cohort are shown in Table 1. There were 712 men (63.80%) and 404 women (36.20%) in the AKI group, and 4,794 males (62.66%) and 2857 females (37.34%) in the non-AKI group (we noted no statistical differences between the sexes; *p* = 0.46). Our analysis of age showed that the highest incidence was in the elderly group (66 years or above, 15.92%) relative to the young (18–40 years old, 10.46%) and middle-aged groups (41–65 years old, 10.89%) (*p* < 0.001). Particular attention should be given to the kidney function of elderly patients who use this drug and who experienced favorable prevention. Of the patients included in our study, there was no significant difference in the length of the hospital stay between the two groups (16.95 days vs. 17.06 days, *p* = 0.32), but the hospitalization costs for the AKI group were significantly higher than those for the non-AKI group (101,269.39 yuan vs. 56,175.75 yuan; *p* < 0.001).

**TABLE 1:** Cefoperazone sulbactam sodium related AKI and patient characteristics.

Group	AKI (*n* = 1116)	Non-AKI (*n* = 7651)	Total (*n* = 8767)	χ^2^/Z	*p* value
**Sex**					
Male	712 (63.80%)	4,794 (62.66%)	5,506 (62.80%)	0.54	0.461
Female	404 (36.20%)	2,857 (37.34%)	3,261 (37.20%)		
Age	67 (55,79)	65 (54,76)	66 (54,76)	18,767,274	<0.001
**Age group**					
Youth	139 (12.46%)	1,190 (15.55%)	1,329 (15.16%)	49.12	<0.001
Middle age	449 (40.23%)	3,673 (48.01%)	4,122 (47.02%)		
Old age	528 (47.31%)	2,788 (36.44%)	3,316 (37.82%)		
**Smoking**					
Yes	404 (36.20%)	2578 (33.70%)	2982 (34.01%)	2.72	0.098
No	712 (63.80%)	5073 (66.30%)	5785 (65.99%)		
Length of hospital stay*	16.95 (2.03, 187.11)	17.06 (2.02, 160.66)	17.04 (2.02, 357.1)	4,347,199.50	0.324
Hospital cost*	101,269.39 (8733.58, 1718345.17)	56,175.75 (142.60, 911656.83)	60,424.85 (142.6, 1718345.17)	2,652,208.50	<0.001

*Two sample Wilcoxon rank sum test was used.

AKI, acute kidney injury.

Of the AKI cohort administered mezlocillin-sulbactam sodium, 166 patients (62.64%) were men and 99 patients (37.36%) were women; these proportions did not differ in the group without AKI, with 1760 male patients (67.12%) and 862 female patients (32.88%) (Table 2). Similar to the situation for patients using cefoperazone-sulbactam sodium, the two groups of patients using mezlocillin-sulbactam sodium also exhibited significant differences in age, suggesting that older age was considered a risk factor for AKI. We also noted that compared with non-AKI patients, the hospitalization costs of patients with AKI were higher, and that the length of hospitalization was longer (*p* < 0.001; Table 2).

**TABLE 2:** Mezlocillin sulbactam sodium related AKI and patient characteristics.

**Group**	**AKI (*n* = 265)**	**Non-AKI (*n* = 2622)**	**Total (*n* = 2887)**	**χ** ^**2**^ **/Z**	***p* value**
**Sex**					
Male	166 (62.64%)	1,760 (67.12%)	1,926 (66.71%)	2.18	0.140
Female	99 (37.36%)	862 (32.88%)	961 (33.29%)		
Age	67 (55,79)	65 (54,76)	66 (54,76)	18,767,274	<0.001
**Age group**					
Youth	35 (13.21%)	532 (20.29%)	567 (19.64%)	8.09	0.017
Middle age	144 (54.34%)	1,353 (51.60%)	1,497 (51.85%)		
Old age	86 (32.45%)	737 (28.11%)	823 (28.51%)		
**Smoking**					
Yes	88 (33.21%)	854 (32.57%)	942 (32.63%)	0.04	0.833
No	177 (66.79%)	1768 (67.43%)	1945 (67.37%)		
Length of hospital stay*	14.28 (2.13, 118.09)	14.13 (2.02, 141.68)	14.14 (2.02, 141.68)	327,805.50	0.129
Hospital cost*	67,261.22 (10159.43, 90349.49)	36,758.32 (3205.32, 838505.01)	38,466.87 (3205.32, 903495.49)	237,879	<0.001

*Two sample Wilcoxon rank sum test was used.

AKI, acute kidney injury.

### 3.2 Comorbidities and concomitant therapies

Based on a review of diagnoses and complications found on the first page of inpatient medical records with respect to target drugs, most patients often had one or more underlying diagnoses or multiple disease complications during hospitalization. In the cefoperazone-sulbactam sodium cohort, patients with AKI tended to demonstrate multiple underlying diseases as shown in Table 3. Several basic diseases showed significant differences between the two groups, including hypertension (χ^2^, 42.68; *p* < 0.001), diabetes (χ^2^, 36.79; *p* < 0.001), cerebral apoplexy (χ^2^, 118.13; *p* < 0.001), anemia (χ^2^, 75.40; *p* < 0.001), coronary heart disease (χ^2^, 72.86; *p* < 0.001), pneumonia (χ^2^, 239.13; *p* < 0.001), sepsis (χ^2^, 101.42; *p* < 0.001), heart failure (χ^2^, 173.38; *p* < 0.001), neoplastic diseases (χ^2^, 31.17; *p* < 0.001), chronic renal insufficiency (χ^2^, 39.93; *p* < 0.001), hypokalemia (χ^2^, 13.32; *p* < 0.001), and hyponatremia (χ^2^, 16.62; *p* < 0.001). The application of several combined medications also produced significant difference between different groups, including nonsteroidal anti-inflammatory drugs (NSAID) (χ^2^, 13.23; *p* < 0.001), angiotensin II receptor inhibitor (ARB) drugs (χ^2^, 4.70; *p* < 0.001), proton pump inhibitors (PPIs) (χ^2^, 59.24; *p* < 0.001), aminoglycosides (χ^2^, 10.38; *p* < 0.001), and diuretics (χ^2^, 391.02; *p* < 0.001).

**TABLE 3:** Comorbidities and concomitant therapies of cefoperazone-sulbactam sodium-related AKI.

	AKI (*n* = 1116)	Non-AKI (*n* = 7651)	Total (*n* = 8767)	χ^2^	*p* value
Comorbidities					
Hypertension	573 (51.34%)	3137 (41.00%)	3710 (42.32%)	42.68	<0.001
Diabetes	336 (30.11%)	1678 (21.93%)	2014 (22.97%)	36.79	<0.001
Cerebral apoplexy	499 (44.71%)	2192 (28.65%)	2691 (30.69%)	118.13	<0.001
Anemia	201 (18.01%)	724 (9.46%)	925 (10.55%)	75.40	<0.001
Coronary heart disease	424 (37.99%)	1974 (25.80%)	2398 (27.35%)	72.86	<0.001
Pneumonia	509 (45.61%)	1816 (23.74%)	2325 (26.52%)	239.13	<0.001
Shock	221 (19.80%)	242 (3.16%)	463 (5.28%)	539.10	2.975
Sepsis	41 (3.67%)	42 (0.55%)	83 (0.95%)	101.42	<0.001
Heart failure	182 (16.31%)	427 (5.58%)	609 (6.95%)	173.38	<0.001
Skin tissue infection	10 (0.90%)	58 (0.76%)	68 (0.77%)	0.24	0.624
Neoplastic disease	200 (17.92%)	1961 (25.63%)	2161 (24.65%)	31.17	<0.001
Gout	14 (1.25%)	50 (0.65%)	64 (0.73%)	4.85	0.027
Chronic renal insufficiency	72 (6.45%)	217 (2.84%)	289 (3.30%)	39.93	<0.001
Pancreatitis	35 (3.14%)	276 (3.61%)	311 (3.55%)	0.63	0.427
COPD	38 (3.41%)	186 (2.43%)	224 (2.56%)	3.71	0.054
Hypokalemia	75 (6.72%)	327 (4.27%)	402 (4.59%)	13.32	<0.001
Hyponatremia	60 (5.38%)	232 (3.03%)	292 (3.33%)	16.62	<0.001
Cirrhosis	54 (4.84%)	379 (4.95%)	433 (4.94%)	0.03	0.869
Hyperlipidemia	28 (2.51%)	141 (1.84%)	169 (1.93%)	2.29	0.131
Combination therapy					
NSAIDs	405 (36.29%)	2362 (30.87%)	2767 (31.56%)	13.23	<0.001
ARB	196 (17.56%)	1152 (15.06%)	1348 (15.38%)	4.70	0.031
ACEI	74 (6.63%)	414 (5.41%)	488 (5.57%)	2.76	0.096
PPI	961 (86.115)	1856 (24.26%)	2817 (32.13%)	59.24	<0.001
Aminoglycoside	162 (14.52%)	1414 (18.48%)	1576 (17.98%)	10.38	<0.001
Diuretics	1005 (90.05%)	3096 (40.47%)	4101 (46.78%)	391.02	<0.001

AKI, acute kidney injury; COPD, chronic obstructive pulmonary disease; NSAIDs, nonsteroidal anti-inflammatory drugs; ARB, angiotensin II receptor inhibitor drugs; ACEI, angiotensin-converting enzyme inhibitor; PPI, proton pump inhibitor.

In the total study population of the mezlocillin-sulbactam sodium cohort, a majority of patients showed one or more underlying diseases or multiple disease complications during hospitalization (as shown in Table 4). We thus noted that diabetes (χ^2^, 4.65; *p* < 0.031), cerebral apoplexy (χ^2^, 7.77; *p* = 0.005), anemia (χ^2^, 5.35; *p* < 0.020), coronary heart disease (χ^2^, 14.84; *p* < 0.001), pneumonia (χ^2^, 24.88; *p* < 0.001), shock (χ^2^, 91.14; *p* < 0.001), heart failure (χ^2^, 15.12; *p* < 0.001), chronic renal insufficiency (χ^2^, 4.31; *p* = 0.031), pancreatitis (χ^2^, 3.54; *p* = 0.049), and hypokalemia (χ^2^, 8.11; *p* < 0.001) constituted adverse factors that affected the occurrence of AKI in patients. Patients with such diseases were often more likely to have had AKI. Similarly, several combined medications also exhibited significant differences, including NSAIDs (χ^2^, 5.31; *p* < 0.021), angiotensin-converting enzyme inhibitor (ACEI) drugs (χ^2^, 7.11; *p* = 0.007), PPIs (χ^2^, 33.24; *p* < 0.001), aminoglycosides (χ^2^, 33.01; *p* < 0.001), and diuretics (χ^2^, 88.04; *p* < 0.001).

**TABLE 4:** Comorbidities and concomitant therapies for mezlocillin-sulbactam sodium-related AKI.

	AKI (*n* = 265)	Non-AKI (*n* = 2622)	Total (*n* = 2887)	χ^2^	*p* value
Comorbidities					
Hypertension	110 (41.50%)	1014 (38.67%)	1124 (38.93%)	0.81	0.367
Diabetes	62 (23.40%)	472 (18.00%)	534 (18.50%)	4.65	0.031
Cerebral apoplexy	72 (27.17%)	522 (19.91%)	594 (20.57%)	7.77	0.005
Anemia	26 (9.81%)	161 (6.14%)	187 (6.48%)	5.35	0.020
Coronary heart disease	72 (27.17%)	460 (17.54%)	532 (18.43%)	14.84	<0.001
Pneumonia	91 (34.34%)	550 (21.05%)	641 (22.20%)	24.88	<0.001
Shock	34 (12.83%)	56 (2.13%)	90 (3.12%)	91.14	<0.001
Heart failure	18 (6.80%)	67 (2.56%)	85 (2.94%)	15.12	<0.001
Neoplastic disease	65 (24.53%)	688 (26.24%)	753 (26.08%)	0.37	0.545
Chronic renal insufficiency	11 (4.15%)	56 (2.14%)	67 (2.32%)	4.31	0.037
Pancreatitis	5 (1.89%)	20 (0.76%)	25 (0.87%)	3.54	0.049
COPD	7 (2.64%)	74 (2.82%)	81 (2.81%)	0.03	0.865
Hypokalemia	15 (5.66%)	68 (2.59%)	83 (2.87%)	8.11	<0.001
Hyponatremia	8 (3.02%)	62 (2.35%)	70 (0.24%)	0.44	0.509
Cirrhosis	8 (3.02%)	66 (2.52%)	74 (2.56%)	0.24	0.622
Hyperlipidemia	1 (0.38%)	26 (0.99%)	27 (0.94%)	0.05	0.827
Combination therapy					
NSAIDs	72 (27.17%)	552 (21.05%)	624 (21.61%)	5.31	0.021
ARB	28 (10.57%)	293 (11.17%)	321 (11.11%)	0.09	0.764
ACEI	14 (5.28%)	65 (2.48%)	79 (2.74%)	7.11	0.007
PPI	72 (27.17%)	1426 (54.39%)	1498 (51.89%)	33.24	<0.001
Aminoglycoside	55 (20.75%)	1013 (38.63%)	1068 (36.99%)	33.01	<0.001
Diuretics	190 (71.70%)	1092 (41.65%)	1282 (44.41%)	88.04	<0.001

AKI, acute kidney injury; COPD, chronic obstructive pulmonary disease; NSAIDs, nonsteroidal anti-inflammatory drugs; ARB, angiotensin II receptor inhibitor drugs; ACEI, angiotensin-converting enzyme inhibitor; PPI, proton pump inhibitor.

### 3.3 Relationships between AKI risk and other laboratory indicators

There are several common indicators that reflect kidney function in clinical practice, including sCr, uric acid, and bilirubin. We collected effective clinical indicators from patients in the AKI and non-AKI groups to evaluate the relationship between changes in kidney function and target drug use. As shown in Supplementary Tables S2, S3, several indicators manifested significant difference between AKI and non-AKI groups after using cefoperazone-sulbactam sodium—including sCr, white blood cell count, red blood cell count, platelet count, β-2 microglobulin, total bilirubin, and uric acid. After using mezlocillin-sulbactam sodium, the majority of the kidney function indicators showed significant differences between the two groups, except for uric acid.

### 3.4 Multivariate logistic regression analysis

A multiple logistic regression analysis of risk factors was additionally performed based on the variables with significant differences between the AKI and non-AKI groups. As shown in Table 5, in the cefoperazone-sulbactam sodium cohort several risk factors showed significant association with AKI in hospitalized patients, including cerebral apoplexy (OR, 1.39; 95% CI, 1.16–1.66), pneumonia (OR, 1.60; 95% CI, 1.35–1.90), sepsis (OR, 2.29; 95% CI, 1.25–4.16), heart failure (OR, 1.52; 95% CI, 1.17–1.95), combined use of PPI (OR, 1.41; 95% CI, 1.13–1.76), and the combined use of diuretics (OR, 3.29; 95% CI, 2.56–4.28). In the mezlocillin-sulbactam sodium cohort, the risk factors showing a significant association with AKI in hospitalized patients included age (OR, 0.45; 95% CI, 0.27–0.71), combined use of aminoglycosides (OR, 0.48; 95% CI, 0.33–0.68), combined use of diuretics (OR, 1.91; 95% CI, 1.33–2.74), and continuous renal replacement therapy (OR, 4.77; 95% CI, 1.14–25.26) (Table 6).

**TABLE 5:** Multivariate logistic regression analysis of AKI associated with cefoperazone and sulbactam sodium in inpatients.

Variable	β	Wald value	OR and 95% CI	*p* value
Age	0.14	2.07	1.15 (0.95–1.38)	0.151
Comorbidity				
Cerebral apoplexy	0.33	13.69	1.39 (1.16–1.66)	<0.001
Anemia	0.06	0.24	1.06 (0.83–1.33)	0.621
Coronary heart disease	0.02	0.04	1.02 (0.83–1.24)	0.837
Pneumonia	0.47	29.98	1.60 (1.35–1.90)	<0.001
Sepsis	0.83	7.47	2.29 (1.25–4.16)	0.006
Heart failure	0.42	10.37	1.52 (1.17–1.95)	0.001
Neoplastic disease	−0.07	0.43	0.93 (0.76–1.14)	0.512
Hyponatremia	0.16	0.64	1.17 (0.78–1.71)	0.425
Combination therapy				
NSAIDs	−0.16	2.83	0.85 (0.71–1.02)	0.092
ARB	−0.15	1.90	0.85 (0.68–1.06)	0.168
Aminoglycosides	−0.02	0.03	0.98 (0.79–1.21)	0.870
PPI	0.35	9.57	1.41 (1.13–1.76)	0.002
Diuretics	1.19	82.61	3.29 (2.56–4.28)	<0.001
Laboratory Values				
sCr	0.01	85.93	1.01 (1.00–1.01)	<0.001
White blood cell count	0.04	47.83	1.04 (1.02–1.05)	<0.001
Red blood cell count	−0.18	11.87	0.83 (0.75–0.92)	0.001
Platelet count	−0.01	19.27	0.98 (0.97–1.00)	<0.001
β-2 microglobulin	0.02	4.25	1.01 (1.00–1.03)	0.039
Total bilirubin	0.01	14.76	1.01 (1.00–1.04)	<0.001
Intervention				
CRRT	1.76	37.53	5.82 (3.35–10.38)	<0.001

NSAIDs, nonsteroidal anti-inflammatory drugs; ARB, angiotensin II receptor inhibitor drugs; PPI, proton pump inhibitor; sCr, serum creatinine; CRRT, continuous renal replacement therapy.

**TABLE 6:** Multivariate logistic regression analysis of AKI associated with mezlocillin sulbactam sodium in inpatients.

Variable	β	Wald value	OR and 95% CI	*p* value
Age	−0.80	10.87	0.45 (0.27–0.71)	0.001
Comorbidity				
Coronary heart disease	−0.03	0.02	0.96 (0.64–1.43)	0.879
Pneumonia	0.14	0.59	1.14 (0.80–1.61)	0.442
Shock	0.02	0.04	1.02 (0.83–1.24)	0.837
Anemia	−0.05	0.02	0.95 (0.45–1.87)	0.899
Heart failure	−0.51	1.34	0.59 (0.23–1.36)	0.247
Chronic renal insufficiency	0.29	0.61	1.34 (0.61–2.72)	0.436
Hypokalemia	0.92	2.76	2.52 (0.76–7.00)	0.096
Pancreatitis	−0.03	0.02	0.96 (0.64–1.43)	0.879
Combination therapy				
NSAIDs	−0.04	0.04	0.96 (0.65–1.38)	0.835
ACEI	0.42	1.38	1.52 (0.72–2.98)	0.240
Aminoglycosides	−0.73	15.80	0.48 (0.33–0.68)	<0.001
PPI	0.21	1.41	1.23 (0.87–1.75)	0.235
Diuretics	0.65	12.51	1.91 (1.33–2.74)	<0.001
Intervention				
CRRT	1.56	4.15	4.77 (1.14–25.26)	0.042
Mechanical ventilation	0.67	8.55	1.95 (1.23–3.04)	0.003
Laboratory Values				
White blood cell count	0.03	4.63	1.02 (1.00–1.05)	0.031
Red blood cell count	0.05	0.29	1.05 (0.87–1.27)	0.587
β-2 microglobulin	0.05	9.87	1.05 (1.02–1.08)	0.002
Platelet count	−0.01	2.90	0.99 (0.9–1.00)	0.089
Total bilirubin	0.01	0.01	1.00 (0.99–1.00)	0.953

AKI, acute kidney injury; NSAIDs, nonsteroidal anti-inflammatory drugs; ACEI, angiotensin-converting enzyme inhibitor; PPI, proton pump inhibitor; CRRT, continuous renal replacement therapy.

### 3.5 Model building

**FIGURE 1:**
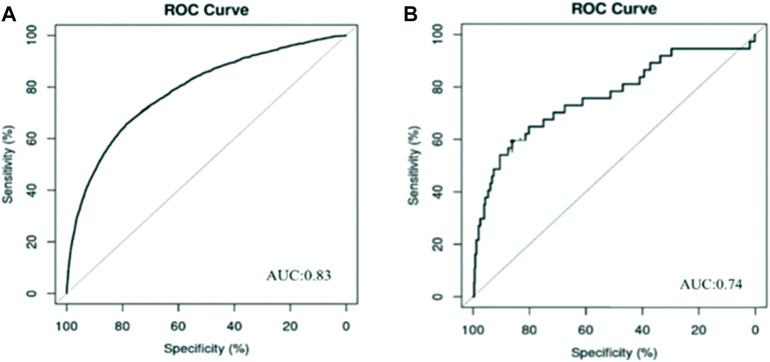
Prediction model of AKI associated with cefoperazone and sulbactam sodium and mezlocillin and sulbactam sodium. **(A)** Cefoperazone-sulbactam sodium; **(B)** Mezlocillin-sulbactam sodium Risk factors analysis and predictive model development of acute kidney injury in inpatients with cefoperazone-sulbactam sodium and mezlocillin-sulbactam sodium: a single-center retrospective study.

The ROC curve for our model with 10-fold CV is illustrated in Figures 1A for all patients treated with cefoperazone and sulbactam sodium. The curve provided a favorable AUC value of 0.83 (95% CI, 0.82–0.84), with a specificity and sensitivity of 76.1% and 74.7%, respectively. The ROC curve for the model with 10-fold CV is shown in Figures 1B for the mezlocillin-sulbactam sodium group, and it provided an AUC value of 0.74 (95% CI, 0.71–0.77), with a specificity and sensitivity of 74.2% and 71.7%, respectively.

## 4 Discussion

The incidence of AKI in hospitalized patients using cefoperazone-sulbactam sodium in the present study was 12.73%, while the rate in hospitalized patients using mezlocillin-sulbactam sodium was 9.18%. These results were higher than the 2.0% reported by the First Hospital of Peking University in 2015 (Yang et al., 2015), but equivalent to the 11.6% reported by Southern Medical University in 2013 (Fang et al., 2010). The high incidence rate of acute kidney injury caused by cefoperazone-sulbactam sodium may be related to its wide application and combination of drugs. This was consistent with the conclusions of previous studies. In our previous study, we demonstrated that the incidence of AKI associated with diuretics was significantly higher than with the aforementioned two drugs at 14.26% (2589/18,148) (Zhang et al., 2022), which may be related to the large number of diuretic drugs used at our hospital. Although the clinical information in the previously published and current studies was collected from the same center (i.e., the First Affiliated Hospital of Shandong First Medical University), the populations were different between the two studies. The populations in the previous work were inpatients who received diuretics, while in the present study they were inpatients receiving cefoperazone-sulbactam or mezlocillin-sulbactam. We are therefore confident that these two studies reflect disparate populations. In a retrospective study conducted in a rural Ethiopian hospital (Riley et al., 2013), the authors discerned an AKI incidence of 20% during routine clinical care using the Acute Kidney Injury Network (AKIN) definition of AKI (Bellomo et al., 2004). Over 13 million people still experience AKI annually worldwide, and more than 1.7 million people have died from this disease (Riley et al., 2013), with a mortality rate as high as 50%–80% (Li et al., 2013) in patients with AKI who need dialysis. This phenomenon might reflect the tremendous investitures of time and effort in AKI by different countries and regions worldwide, with the economic pressure on developing countries and regions being particularly prominent.

Recently, Hodgson et al. summarized the efforts of predictive models and AKI alerts reported over recent decades and concluded that the early diagnosis of AKI can only improve outcomes when adequate interventions follow (Kurzhagen et al., 2020). Among the inpatients diagnosed with AKI in our cohort receiving cefoperazone-sulbactam sodium, the elderly group accounted for a large proportion of the entire AKI group (47.31%), while in the mezlocillin-sulbactam sodium cohort, middle-aged patients also accounted for a large proportion (54.34%). These data indicate that older individuals are more likely to develop AKI than younger individuals, potentially because elderly and middle-aged patients often possess multiple underlying diseases or complications during their hospitalization. Moreover, elderly patients often use multiple drugs simultaneously during hospitalization (Formica et al., 2018), increasing kidney burden. In contradistinction, the effects could be related to the physiologic decline in organ function, lower kidney reserve function, and lower compensatory capacity in elderly patients (Nash et al., 2002; Xue et al., 2006). The results from both cohorts in our study showed that patients in the AKI group exhibited longer hospital stays than those in the non-AKI group, and that the hospital expenses were also relatively high. These results may be due to the fact that patients with AKI are often more seriously ill, and thus the length of hospital stay is usually longer.

In addition to the effect of drugs on the kidney, the condition of the patients’ underlying diseases during hospitalization and the condition of the patients’ combined medication during hospitalization will also affect the diagnosis and prognosis of patients with AKI. Among the risk factors that induce AKI in patients, the combination of one or more underlying diseases, or the occurrence of complications during the treatment process are unfavorable for the occurrence and development of acute kidney injury. In our study, patients with hypertension, diabetes, coronary heart disease, heart failure, tumor disease and chronic kidney disease have adverse effects on patients with acute kidney injury caused by target drugs. Congruent with the literature (Pierson-Marchandise et al., 2017), the two drugs we suspected to be associated with AKI risk and analyzed in this study showed that the risk of AKI induction was particularly high in a multi-drug environment and exhibited commensurate increases with the increase in the number of prescription drugs. Cefoperazone-sulbactam sodium may have elevated the risk of AKI in patients with stroke, pneumonia, sepsis, heart failure, or other diseases. In current worldwide epidemiologic surveys conducted on AKI, although most authors have investigated the disease from a macro perspective—elucidating its epidemiologic characteristics and analyzing the risk factors leading to the disease—some specific groups have often been ignored (Dinh et al., 2021). Our results showed that there was a significant difference in kidney function indicators between AKI and non-AKI groups, and we perceived that after the use of target drugs the AKI group patients often manifested a significant increase in laboratory indicators compared with the non-AKI group patients. Therefore, it is important to provide early detection and timely intervention in AKI by paying particular attention to the laboratory indicators of kidney function of inpatients with the aforementioned underlying diseases. In addition to the effect of antibacterial drugs on AKI, our study depicted that the combination of aminoglycosides, diuretics, and proton-pump inhibitors increased the risk of AKI during hospitalization. This effect may have been due to the combined use of multiple drugs, changing the hemodynamics of the kidney and resulting in a reduction in glomerular filtration pressure or renal blood flow (or both) (Prieto-Garcia et al., 2016); this, in turn, culminated in a diminution in GFR, further augmenting the risk of AKI in patients. In previous studies, cephalosporins-related acute kidney injury accounted for 93.04% of all β lactams drugs. The main reason for the occurrence of acute kidney injury is that its metabolites accumulate in cells when excreted by the kidneys, resulting in nephrotoxicity (Bentley et al., 2010). Next are penicillins, which generally do not have direct nephrotoxicity. It causes acute interstitial nephritis by causing allergic reactions, leading to acute kidney injury (Pannu and Nadim, 2008; Bentley et al., 2010). Therefore, with respect to patients with AKI (especially for those individuals administered multiple drugs and possessing underlying disease), we recommend that clinicians give closer attention to enhance patient safety and to optimize pharmacotherapy.

Although there are many AKI-prediction tools offered in previous studies, there are few parallel tools that detect AKI risk in clinical settings. In 2016, Koyner et al. (Koyner et al., 2016) developed a model for AKI risk prediction based on a non-ICU patient cohort, with an AUC ROC of 0.74. However, additional data such as vital signs and laboratory indicators were not included in their study. Our AUC values for the AKI-prediction models associated with cefoperazone-sulbactam sodium and mezlocillin-sulbactam sodium were 0.83 (95% CI, 0.82–0.84) and 0.74 (95% CI, 0.71–0.77), respectively. Thus, compared with previous studies, the performance of our model was superior and the variables included were more extensive.

In our previous study, we evaluated AKI in hospitalized patients who underwent diuretic treatment, while in the present study we focused on two other drugs, cefoperazone-sulbactam sodium and mezlocillin-sulbactam sodium. Since cefoperazone-sulbactam and mezlocillin-sulbactam are widely used to prevent severe infection caused by lactamase microorganisms worldwide, we posit that it would be of great clinical significance to analyze the risk factors involved in AKI and to develop predictive models for AKI in inpatients receiving these medications.

## 5 Limitations of this study

There were several limitations for our study. Firstly, these parameters selected in the study are associated with an increased risk of developing acute kidney injury regardless of the etiology. Therefore, it is difficult to distinguish to what extent this model actually predicts the development of AKI. Besides, in this study, the diagnosis of acute kidney injury in our hospital was mostly completed through blood creatinine. Because in clinical practice, continuous long-term urine volume monitoring of patients is not practical and cannot accurately capture urine volume changes that meet the guidelines for acute kidney injury.

## 6 Conclusion

We herein conducted a single-center retrospective study. The incidence of cefoperazone-sulbactam sodium-related AKI was 12.73% (1116/8767), and the rate for mezlocillin-sulbactam sodium-related AKI was 9.18% (265/2887). We screened out several risk factors for cefoperazone-sulbactam sodium, including the presence of underlying diseases (e.g., cerebral apoplexy, pneumonia, sepsis, and heart failure), and the combined use of PPIs and diuretics. For mezlocillin-sulbactam sodium, several risk factors were also proposed that included older age, combined use of aminoglycosides and diuretics, and the receipt of continuous kidney replacement therapy and mechanical ventilation during hospitalization. Based on the meaningful variables assessed, we developed logistic regression models for AKI risk in hospitalized patients who underwent cefoperazone-sulbactam and mezlocillin-sulbactam sodium treatments. The AUC of the cefoperazone-sulbactam predictive model was 0.83 (95% CI, 0.82–0.84), and the AUC for the predictive model reflecting mezlocillin-sulbactam sodium use was 0.74 (95% CI, 0.71–0.77).

We posit based upon our research results that fully evaluating the risk factors of hospitalized patients before using cefoperazone-sulbactam sodium and mezlocillin-sulbactam sodium would reduce the incidence of AKI. We suggest that it is also necessary to improve the awareness of medical staff and patients regarding AKI.

## Data Availability

The datasets presented in this study can be found in online repositories. The names of the repository/repositories and accession number(s) can be found in the article/Supplementary Material.
