# Supplementary material for: Risk-factor analysis and predictive-model development of acute kidney injury in inpatients administered cefoperazone-sulbactam sodium and mezlocillin-sulbactam sodium: a single-center retrospective study
Source: Front Pharmacol. 2023 Jun 8;14:1170987. doi: 10.3389/fphar.2023.1170987 (PMC10286859; doi:10.3389/fphar.2023.1170987)
Supplement: Supplementary file 1 [file DataSheet1.docx]

**Risk factors analysis and** **predictive model development of acute kidney injury in inpatients with cefoperazone-sulbactam sodium and mezlocillin-sulbactam sodium: a single-center retrospective study**

Ruiqiu Zhang^1^, Liming Gao^1^, Ping Chen^2^, Weiguo Liu^1^, Xin Huang^1^, Xiao Li^1*^

*1. Department of Clinical Pharmacy, The First Affiliated Hospital of Shandong First Medical University & Shandong Provincial Qianfoshan Hospital, Shandong Engineering and Technology Research Center for Pediatric Drug Development, Shandong Medicine and Health Key Laboratory of Clinical Pharmacy, Jinan 250014, China*

*2. Department of Nephrology, The First Affiliated Hospital of Shandong First Medical University & Shandong Provincial Qianfoshan Hospital, Jinan, 250014, China*

**Corresponding author**

Xiao Li

E-mail: [lixiao1688@163.com](mailto:lixiao1688@163.com) or [x.li@sdu.edu.cn](mailto:x.li@sdu.edu.cn)

**Methodology**

**Clinical data**

The data were extracted from the clinical information of the electronic medical records in our center. We included patients who were discharged from hospital from January 1, 2018 to December 31, 2020 and received treatment with cefoperazone-sulbactam sodium and mezlocillin-sulbactam sodium. The patients were excluded if met any of the following criteria: (1) hospital stay < 48h; (2) Age < 18 years; (3) glomerular filtration rate (GFR) < 30ml/min/1.73m2 within 48 hours after admission; (4) AKI was diagnosed on admission; (5) less than two serum creatinine (Scr) test results during hospitalization; (6) the Scr values were always lower than 40 μmol/L during hospitalization; (7) cases with incomplete medical history information.

If no measurement of a clinical variable was available for a given time, a carryforward imputation method was employed to fill the missing measurement with the most recently available previous measurement, a causal procedure. A multidisciplinary research team, including clinical pharmacist, clinician, epidemiologist, and statistician, was developed to ensure effective implementation of the study.

**Statistical Analyses**

All data analysis in this study were conducted through R software (version 3.6.3). Kolmogorov Smirnov (K-S) test was employed to test the normality first for continuous variables. A P value ≥ 0.05 suggested obeying the normal distribution, while P < 0.05 suggested disobeying the normal distribution. The data conforming to the normal distribution was expressed by the mean ± SD, and the difference between groups was tested by t test or analysis of variance. The data that do not conform to the normal distribution are expressed by the median and interquartile interval, and the Wilcoxon rank sum test was used for inter-group comparison. For count data, the number of cases (n) or constituent ratio (%) were used. Chi-square test was used for comparison between groups. The research variable was used as the independent variable, the AKI group was used as the dependent variable, and the chi-square test was used for univariate analysis. Then the variables with p<0.05 in univariate analysis were included in multivariate logistic regression analysis to determine the significant independent risk factors of acute kidney injury caused by aminoglycosides in hospitalized patients. The analysis results were expressed by odds ratio (OR) and 95% confidence interval (95% CI). All p values were bilateral, and p value<0.05 was considered significant.

**Table S1.** Diagnostic criteria of AKI according to KDIGO 2012 guidelines

| AKI can be diagnosed if one of the following criterias is met: | |
| --- | --- |
| 1 | Within 48 hours, the absolute value of serum creatinine (Scr) increased ≥ 0.3mg/dl (26.5 umol/L); |
| 2 | Known or speculated increase of Scr within 7 days ≥ 1.5 times of baseline value; |
| 3 | Urine volume ≤ 0.5ml/kg/h for more than 6h. |

**Table S2 Distribution of laboratory values in patients with AKI and non-AKI associated with cefoperazone and sulbactam sodium**

|  | **AKI**  **(n=1116)** | **Non-AKI**  **(n=7651)** | **c^2^/Z** | ***P* Value** |
| --- | --- | --- | --- | --- |
| Scr | 89.15(16,1383.60) | 64(17,1259) | 2751146.50 | <0.001 |
| White blood cell count | 10.35(0.01,99.94) | 8.47(0.01,288.16) | 3242075 | <0.001 |
| Red blood cell count | 3.19(1.06,6.30) | 3.73(0.62,7.56) | 5254877.50 | <0.001 |
| Platelet count | 176(2,381381) | 216(1,552552) | 4808673.50 | <0.001 |
| β-2 microglobulin | 3.99(0.94,419.74) | 2.27(0.73,168.88) | 2174816.50 | <0.001 |
| Total bilirubin | 12.2(1.40,751.20) | 11.1(0.40,692) | 3701946 | <0.001 |
| Uric acid | 281(21,1108) | 216(21,1077) | 2418042 | <0.001 |

AKI: acute kidney injury

**Table S3 Distribution of laboratory values in patients with AKI and non-AKI associated with mezlocillin sulbactam sodium**

|  | **AKI**  **(n=265)** | **Non-AKI**  **(n=2622)** | **c^2^/Z** | ***P* Value** |
| --- | --- | --- | --- | --- |
| Scr | 84(29,533) | 71(20,691) | 245921.50 | <0.001 |
| White blood cell count | 8.49(2.65,66.91) | 7.82(0.22,56.17) | 264734.50 | <0.001 |
| Red blood cell count | 3.69(1.80,6.26) | 3.98(1.3,7.41) | 364003 | <0.001 |
| platelet count | 206(8,524524) | 225(2,552552) | 364265.50 | 0.018 |
| β- 2 microglobulin | 3.24(1.34,121.02) | 2.24(0.88,72.30) | 193002.50 | <0.001 |
| Total bilirubin | 10.9(1.90,533.40) | 10(0.50,537.30) | 264451 | 0.003 |
| Uric acid | 242(49,1199) | 253(34,838) | 275405.50 | 0.642 |

AKI: acute kidney injury

**Key points**

**Question: Are cefoperazone sulbactam sodium and mezlocillin sulbactam sodium associated with the risk of AKI in hospitalized patients?**

**Findings: Among the inpatients who use cefoperazone and sulbactam sodium and mezlocillin and sulbactam sodium, the combination of multiple drugs may increase the risk of AKI. The AKI** predictive **model based on machine learning has good** predictive **performance in predicting the AKI of adult inpatients who use cefoperazone and sulbactam sodium or mezlocillin and sulbactam sodium.**

**Meaning: The evaluation of risk factors before the use of antibiotics should be helpful to the early prevention, diagnosis and treatment of AKI, and ultimately reduce the incidence rate and improve the prognosis.**
